# Supervised machine learning statistical models for visual outcome prediction in macular hole surgery: a single-surgeon, standardized surgery study

**DOI:** 10.1186/s40942-025-00630-3

**Published:** 2025-01-13

**Authors:** Kanika Godani, Vishma Prabhu, Priyanka Gandhi, Ayushi Choudhary, Shubham Darade, Rupal Kathare, Prathiba Hande, Ramesh Venkatesh

**Affiliations:** 1 Department of Retina and Vitreous, Narayana Nethralaya, #121/C, 1st R Block, Chord Road, Rajaji Nagar, Bengaluru, 560010 India; 2 Narayana Nethralaya, #121/C, Chord Road, 1st R Block Rajaji Nagar, Bangalore, 560010 India

**Keywords:** Macular hole, Outcomes, Machine learning, Statistical models, Prediction

## Abstract

**Purpose:**

To evaluate the predictive accuracy of various machine learning (ML) statistical models in forecasting postoperative visual acuity (VA) outcomes following macular hole (MH) surgery using preoperative optical coherence tomography (OCT) parameters.

**Methods:**

This retrospective study included 158 eyes (151 patients) with full-thickness MHs treated between 2017 and 2023 by the same surgeon and using the same intraoperative surgical technique. Data from electronic medical records and OCT scans were extracted, with OCT-derived qualitative and quantitative MH characteristics recorded. Six supervised ML models—ANCOVA, Random Forest (RF) regression, K-Nearest Neighbor, Support Vector Machine, Extreme Gradient Boosting, and Lasso regression—were trained using an 80:20 training-to-testing split. Model performance was evaluated on an independent testing dataset using the XLSTAT software. In total, the ML statistical models were trained and tested on 14,652 OCT data points from 1332 OCT images.

**Results:**

Overall, 91% achieved MH closure post-surgery, with a median VA gain of -0.3 logMAR units. The RF regression model outperformed other ML models, achieving the lowest mean square error (MSE = 0.038) on internal validation. The most significant predictors of VA were postoperative MH closure status (variable importance = 43.078) and MH area index (21.328). The model accurately predicted the post-operative VA within 0.1, 0.2 and 0.3 logMAR units in 61%, 78%, and 87% of OCT images, respectively.

**Conclusion:**

The RF regression model demonstrated superior predictive accuracy for forecasting postoperative VA, suggesting ML-driven approaches may improve surgical planning and patient counselling by providing reliable insights into expected visual outcomes based on pre-operative OCT features.

**Clinical trial registration number:**

Not applicable.

## Introduction

An idiopathic macular hole (MH) is generally initiated by anteroposterior tractional forces from the posterior cortical vitreous, compounded by tangential forces from the epiretinal membrane (ERM) and internal limiting membrane (ILM) leading to recent-onset visual symptoms, including blurring, metamorphopsia, and mild to moderate vision loss [1, 2]. 

Optical coherence tomography (OCT) is the primary imaging modality utilized in the evaluation of MHs, offering detailed insights into MH dimensions and morphology that are critical for predicting both anatomical and visual outcomes post-surgery [3, 4]. Key MH characteristics, measurements, and indices from OCT are instrumental in forecasting anatomical success, while the integrity of the retinal pigment epithelium (RPE) and photoreceptor layer at the fovea is essential for functional prognosis [4, 5]. Visual improvement following MH surgery largely depends on the restoration of continuity in the outer retinal layers, including the external limiting membrane (ELM) and photoreceptor layer, as well as damage to the outer retinal and RPE layers from surgical trauma or retinal phototoxicity [4, 6]. Consequently, the primary goal of MH surgery should be to achieve a closed MH with reestablishment of the foveal contour and continuity of the outer retinal layers without any damage to the underlying foveal RPE.

Current treatment for MHs involves removal of the tractional forces via pars plana vitrectomy and ILM peeling [1]. This approach has shown anatomic closure success rates ranging between 80% and 95% [7, 8]. However, in a subset of cases, MHs do not close after initial surgery, necessitating a second intervention. Secondary surgeries are generally associated with higher medical costs and less favourable anatomical and functional outcomes. Accurate preoperative assessment is therefore essential to predict the likelihood of MH closure and expected visual acuity (VA) improvement following successful surgery. This can be achieved by analysing specific preoperative clinical features and MH characteristics, especially on OCT.

Predictors of anatomical closure are also likely to influence final visual outcomes. However, most studies on functional outcomes in MH surgery have focused on identifying a single OCT parameter with the greatest impact on surgical success, commonly using linear regression models [4, 9, 10]. This approach, however, may not be feasible for non-linear relationships and interactions between multiple qualitative and quantitative MH features on OCT. A more comprehensive analysis considering the complex interplay of various MH characteristics may provide a more reliable framework for predicting both anatomical and visual outcomes, potentially improving clinical decision-making in MH management.

Artificial intelligence (AI) is being increasingly adopted in healthcare, including in ophthalmology. In the context of MHs, AI applications are emerging across three key areas: diagnosis, identification of MH characteristics, and postoperative prediction of anatomical closure and visual recovery [11–13]. Supervised machine learning (ML), a prominent subset of AI, is particularly effective for developing statistical models or algorithms by analysing a diverse range of qualitative and quantitative independent variables. Recently, ML has shown great promise in outcome predictions. Data mining techniques used to predict clinical outcomes frequently employ various supervised ML algorithms. Through highly repetitive and reproducible mathematical equations, ML can predict both qualitative and quantitative outcomes with significant accuracy. Unlike traditional programming, ML algorithms learn from data, with predictive accuracy improving as more data and variables are incorporated [14, 15]. These developments highlight the potential of AI to contribute substantially to advancing MH care through data-driven, precise predictions and improved clinical decision-making.

Hence, we conducted a comparative analysis of various supervised ML models using OCT-based MH data, with the objective of identifying the model that delivers the highest accuracy and the lowest error in predicting visual outcomes in patients undergoing MH surgery for a single surgeon using the same surgical technique. This approach aims to enhance the precision of outcome predictions, supporting the clinician in both surgical planning and patient counselling.

## Methods

For this study, the hospital’s electronic medical records system was used to identify cases of idiopathic full-thickness MHs that presented to the retina clinic from June 2017 to December 2023. Only patients who underwent MH repair surgery alone without combined phacovitrectomy or additional allied procedures, by the same surgeon (RV) who followed the same intraoperative surgical technique and post-operative instructions, had good-quality pre-operative OCT scans available for OCT features’ documentation and had one follow-up visit at least after one-month post-surgery, with high-quality post-operative OCT scans available for MH closure assessment, were included in the analysis. Exclusion criteria comprised cases of secondary MHs, cases in which surgery was advised but not performed, and those lacking sufficient follow-up data or pre- or post-operative OCT imaging necessary for assessing MH characteristics and closure.

Following the selection of study cases, demographic details (age, gender), ocular information (laterality of the affected eye, pre-operative best-corrected VA, lens status categorized as phakia, pseudophakia, or aphakia), and post-operative VA at least at one-month follow-up were recorded. The presenting and post-operative VA was recorded using Snellen’s scale and later converted to logMAR (logarithm of minimum angle of resolution) for analysis.

All pre-operative OCT scans were acquired with the spectral domain Spectralis (Heidelberg Engineering, Germany) system using the radial line scan protocol, which passed through the foveal center. Each eye underwent 12 or 24 radial OCT scans, depending on the chosen subprotocol. Any scans of poor quality or those failing to pass through the foveal center, thereby not visualizing MH features, were excluded from further analysis. The following pre-operative OCT characteristics were documented and measured for each eligible scan: (1) attachment or detachment of the posterior cortical vitreous over the fovea; (2) presence of ERM adjacent to the MH; (3) presence of intraretinal cystic spaces at the MH edges; (4) RPE hyperreflectivity at the fovea; (5) minimum inner diameter of the MH; (6) maximal basal diameter of the MH; (7) nasal and temporal slopes of the MH; (8) minimum and maximum MH height (in microns); and (9) MH area (in square millimetres). These measurements were obtained using the Spectralis software’s line and area measurement tools (HEYEX version 1.10.2.0, Heidelberg Engineering), with calculated MH indices derived from previously established formulas [16]. 

To ensure reliable documentation of MH measurements, two independent graders (KG and RK) were each assigned a random subset of 30 OCT images from the study dataset. Measurements of MH base diameter, maximum height, and MH area made by the two graders were compared, with inter-rater reliability assessed using Bland-Altman plots. Once intergrader agreement was confirmed with minimal bias, each grader independently analyzed the remaining OCT scans.

The decision to perform MH surgery was at the discretion of the operating surgeon. All patients underwent 25-gauge microincision pars plana vitrectomy with complete induction of posterior vitreous detachment. This was followed by conventional circular ILM peeling around the MH, extending from one retinal arcade to the other. ERM peeling was performed when indicated. Long-acting gas endotamponade (20% SF₆ or 15% C₃F₈) was utilized in all cases, and patients were instructed to maintain a face-down position for a minimum of 7 days postoperatively, regardless of the MH size or the specific endotamponade agent used.

After a minimum follow-up period of 1-month post-surgery, post-operative OCT scans were evaluated to determine MH closure status. Based on Kang’s classification [17], post-surgery outcomes were categorized as: 1) Closed MH, indicating no foveal neurosensory retina defect, or 2) Open MH, where a foveal neurosensory retina defect persisted despite surgery. For each eye, if post-operative OCT showed a ‘closed’ status, the post-operative outcomes for all the pre-operative OCT scans for that eye were classified accordingly; likewise, if the MH remained open, the pre-operative scans were categorized as ‘open.’ The change in the VA after surgery (in logMAR units) was calculated by subtracting the post-operative BCVA (in logMAR units) from the pre-operative BCVA (in logMAR units).

This study was presented to the Institutional Research Board and Ethics Committee and was exempted from further approvals (EC-002/01/2024).

### Data processing

Three distinct data sheets were generated:

1. Patient Information Sheet – containing patient demographics, including age and gender.
2. Ocular Information Sheet – documenting ocular specifics, such as affected eye laterality, pre- and post-operative VA and lens status.
3. Image Information Sheet – detailing both qualitative findings and quantitative measurements of each pre-operative MH as observed on individual OCT images, along with the post-operative MH and outcome.

Any rows or columns with missing values in these sheets were removed to ensure data completeness for analysis. The Image Information Sheet included categorical data, which was encoded using a label encoding technique to facilitate analysis.

### Computational methods

#### Training of different ML statistical models

The data from the Image Information Sheet was randomly divided into a training set and a testing set in an 80:20 ratio. The training dataset, which included both qualitative and quantitative variables, was used to train six ML predictive models with the goal of forecasting a quantitative dependent variable—post-operative VA following MH surgery. The qualitative independent variables on OCT were prefoveal status of the posterior cortical vitreous, presence of ERM, intraretinal cystic spaces, RPE hyperreflectivity at the fovea and post-operative MH status, and the quantitative independent variables were maximal outer diameter, MH area, and other MH indices such as hole forming factor, MH index, tractional hole index and diameter hole index. The analysis was performed using the EasyFit function in XLSTAT software, applying the following models: ANCOVA, RF regression model, K-Nearest Neighbor, SVM, Extreme Gradient Boosting, and Lasso regression model. The model with the highest accuracy and lowest error was selected as the best-performing statistical model for predicting VA outcomes after MH surgery.

#### Validation of the model with a separate testing dataset

The best-performing model was then applied to predict VA outcomes on an independent test set using the EasyPredict function in XLSTAT software. This test set comprised data samples that had not been used during the training phase, ensuring an unbiased evaluation of the model’s predictive performance. The model’s predictions on the testing set were recorded and assessed for accuracy and reliability using performance evaluation metrics available within the XLSTAT model performance indicator tool.

### Statistical analysis

Descriptive statistical analysis for this study was conducted using GraphPad Prism (v10.4.0, GraphPad Software, California, USA). Data distribution was evaluated using the Shapiro-Wilk test for normality. Categorical variables were summarized as frequencies and percentages, while quantitative variables were expressed as medians with interquartile ranges (IQR) or means with standard deviations, as appropriate. The Mann-Whitney U test was utilized to compare unpaired quantitative data, and Fisher’s exact test was used for comparisons of categorical variables between independent groups. Predictive analysis using ML statistical algorithms was performed with XLSTAT (v2023.3.1.1416, Lumivero, Denver, USA) integrated with Microsoft Excel 2021, to assess post-operative VA outcomes following MH surgery.

## Results

The study included 158 eyes who had been diagnosed and operated on for full-thickness MH. The study included 151 patients, 60% (*n* = 90) of whom were females, with an average age of 65.26 ± 6.585 years. 77 (49%) of the 158 MHs affected the right eye. 57 (36%) of the 158 eyes were phakic and the remaining 101 (64%) eyes were pseudophakic. The median pre-operative logMAR VA for the study eyes was 0.78 (20/120), with an IQR of 0.60 to 1.00. After surgery, a MH was closed in 144 (91%) eyes, with a post-operative median logMAR VA of 0.48 (IQR: 0.18–0.70).

1665 high-quality pre-operative OCT images with 18,315 OCT data points provided qualitative and quantitative information about the MH’s characteristics and dimensions. The training set for the various prediction models included information from 1332 (80%) OCT images, while the remaining 333 (20%) OCT images were used to test previously trained models. Table 1 summarises the data gathered from the training and testing sets’ pre-operative OCT images. All MH OCT parameters were comparable between the training and testing datasets.

**Table 1:** Comparisons of the optical coherence tomography parameters between the training and testing datasets

OCT parameter	Training set (*n* = 1332)	Testing set (*n* = 333)	*P* value
Outer diameter (microns) (Mean, SD)	1006 ± 346.1	1039 ± 409.4	0.422
MHA (mm^2^) (Mean, SD)	0.281 ± 0.118	0.297 ± 0.136	0.215
HFF (Mean, SD)	0.754 ± 0.168	0.756 ± 0.209	0.693
MHI (Mean, SD)	0.958 ± 0.873	0.990 ± 0.966	0.665
THI (Mean, SD)	0.249 ± 0.622	0.190 ± 0.515	0.089
DHI (Mean, SD)	0.102 ± 0.211	0.083 ± 0.187	0.283
Prefoveal PVD (n, %)	1087 (82)	285 (86)	0.092
ERM (n, %)	435 (33)	122 (37)	0.173
Intraretinal cystic spaces (n, %)	1302 (98)	326 (98)	> 0.999
RPE hyperreflectivity at the fovea (n, %)	1244 (94)	313 (94)	0.804
Post-operative MH status - Closed (n, %)	1190 (89)	297 (89)	> 0.999
Post-operative MH status - Open (n, %)	142 (11)	36 (11)	

Abbreviations: OCT – optical coherence tomography, SD – standard deviation, HFF – hole forming factor, MHI – macular hole index, THI – tractional hole index, DHI – diameter hole index, PVD – posterior vitreous detachment, ERM – epiretinal membrane, RPE – retinal pigment epithelium, MHA – macular hole area, mm^2^ – square millimetres

**Fig. 1:**
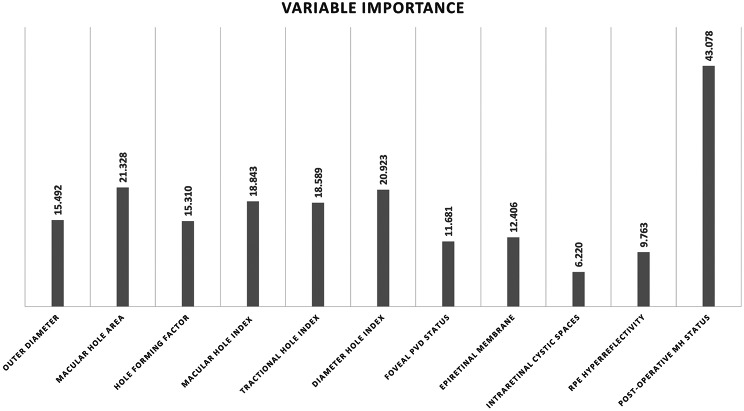
Variable importance for predicting the visual outcome following macular hole surgery with the random forest regression model

The training set data (*n* = 1332 OCT images, 14652 OCT data points) was randomly divided into a separate learning set and an internal validation set at a ratio of 80:20 by the EasyFit function available on the XLSTAT software itself. The learning set consisted of 1066 OCT images, while the internal validation set had 266 OCT images. After training with the learning dataset and analysing the performance metrics of different predictive models on the internal validation dataset, a summary table was created to identify the most accurate model with the least mean square error [MSE] **(**Table 2**)**. The best model, according to the statistic MSE computed on the internal validation sample, was the RF regression model (MSE = 0.038). It outperformed other models in forecasting the VA outcome following a MH surgery. Also, the RF model employed the out-of-bag (OOB) evaluation for internal cross-validation, treating the training set as a test set for quality assessment. The MSE for the OOB sample used for internal cross validation was 0.043. In addition, the best-performing model for our dataset i.e., the RF regression model identified the post-operative MH status as the most critical variable (variable importance = 43.078) followed by MH area index (variable importance = 21.328) for predicting the post-operative VA following MH surgery (Fig. 1).

**Table 2:** Performance metrics of different machine learning algorithms on the internal validation dataset

Performance metrics	ANCOVA	Random forests	Extreme Gradient Boosting	K Nearest Neighbors	SVM	LASSO regression
MAE	0.191	0.149	0.158	0.259	0.217	0.305
MSE	0.057	0.038	0.045	0.112	0.071	0.146
R²	0.491	0.658	0.601	0.001	0.368	-0.303
Adjusted R²	0.479	0.650	0.592	-0.022	0.353	-0.333
Akaike’s AIC	-750.06	-856.19	-814.75	-570.67	-692.40	-500.05
Schwarz’s SBC	-728.55	-834.69	-793.25	-549.17	-670.90	-478.55

Abbreviations: ANCOVA – analysis of covariance, SVM – Support Vector Machine, MAE - mean absolute error, MSE – mean square error, AIC - Akaike information criterion, R – correlation co-efficient, SIC - Schwarz information criterion

**Fig. 2:**
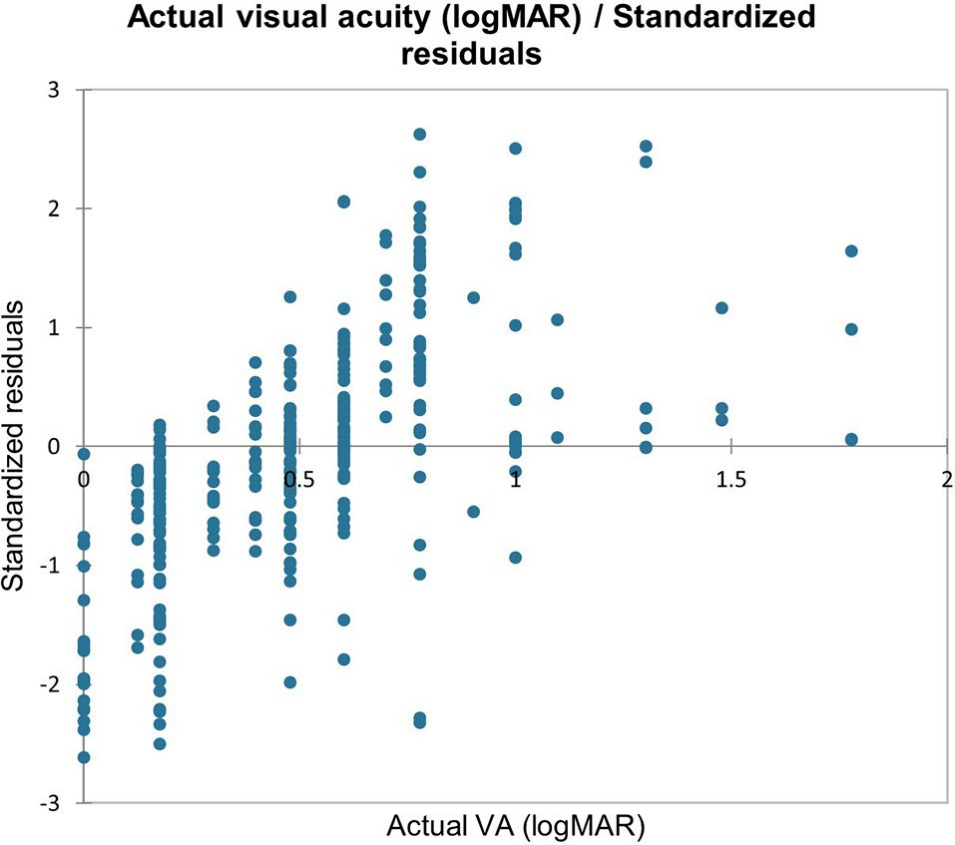
Actual post-operative visual acuity versus standardized residuals

The EasyPredict function was employed to estimate post-operative VA outcomes using a separate testing dataset (*n* = 266 OCT images, 2926 OCT data points) of OCT parameters, applying the RF regression model. The model’s performance was evaluated by calculating the MSE, which was 0.042 when comparing predicted post-operative logMAR VA with actual post-operative logMAR VA. Figure 2 presents a plot of actual post-operative VA versus standardized residuals. The model demonstrated predictive accuracy within 0.1 logMAR units one-line of actual VA in 61% (202/333) of OCT images, within 0.2 logMAR units in 78% (260/333) of OCT images, and within 0.3 logMAR units in 87% (289/333) of OCT images.

## Discussion

In summary, this study evaluated 158 MH eyes operated by a single surgeon and using a common surgical technique, employing preoperative OCT parameters to predict VA outcomes using ML models. Post-surgery, 91% of eyes achieved MH closure, with a median VA change of -0.3 logMAR units. Among the models tested, the RF regression model demonstrated the highest predictive accuracy, outperforming other ML algorithms. The primary predictive factor was postoperative closed MH status. On external testing, the RF model accurately predicted VA within 0.1 logMAR units in 61%, 0.2 logMAR units in 78%, and 0.3 logMAR units in 87% of cases, showing strong potential for clinical application in outcome prognostication.

Maximal improvement in VA following MH surgery depends on achieving a normal-appearing foveal contour and continuous outer retinal layers, which can be supported by a successfully closed MH and a healthy, functioning RPE and outer retina. Key preoperative factors influencing the visual outcome include patient demographics, MH duration, and MH characteristics. Intraoperative variables, such as surgeon skill, intraoperative dye use, and the duration and technique of ILM peeling, also play roles but appear to be less predictive of postoperative outcomes [18–22]. Longer surgical durations with intraoperative dyes, even safe options like Brilliant Blue G (BBG), increase phototoxic risk to the RPE and outer retina [23, 24]. However, recent improvements in ILM visibility and instrumentation have reduced peeling duration, mitigating this risk. While prior studies suggest that surgeon experience minimally impacts outcomes in academic settings, ILM peeling variations designed to achieve anatomical closure in large MHs have produced uncertain effects on VA [25, 26]. Consequently, this study focused on preoperative OCT parameters—such as MH dimensions and indices—rather than intraoperative factors to predict VA outcomes, as preoperative MH characteristics are established predictors of surgical success.

The role of AI in managing MHs has been delineated into different stages of care: diagnosis, identification of MH characteristics, and postoperative predictions concerning hole closure and visual recovery [11]. Although AI is frequently employed in predicting anatomical closure post-surgery, its application in forecasting visual outcomes remains limited [10, 12, 13, 27–29]. Current research predominantly utilizes deep learning methodologies, with the construction of complex artificial neural networks being the standard approach [12, 13, 30, 31]. In contrast, ML techniques have been applied infrequently within the context of MH management [27]. Nevertheless, ML algorithms present several advantages that warrant further exploration. Firstly, ML models exhibit the ability to adjust hyperparameters, enhancing alignment with specific objectives. Secondly, ensemble ML methods offer consistent, reliable, and comprehensive performance. Notably, advanced ML models eliminate the necessity for a collinearity diagnosis among independent variables, which is a requirement for traditional linear regression models [32]. In light of these advantages, this study adopts supervised ML-based predictive statistical models to analyse pre-operative OCT parameters and to forecast the visual outcomes following MH surgery.

In this study, the RF regression model outperformed all other models, demonstrating superior predictive accuracy. Among supervised ML techniques, the RF algorithm is widely adopted for its versatility and ability to combine outputs from multiple decision trees into a single, reliable prediction, making it suitable for both classification and regression tasks [33]. Despite the inclusion of numerous OCT variables, the RF model displayed exceptional accuracy, attributed to its robustness and stability. In contrast, other predictive algorithms, including ANCOVA, Extreme Gradient Boosting, SVM, K-Nearest Neighbor, and LASSO regression, showed higher MSEs, indicating lower predictive accuracy in this study. This discrepancy may be due to the challenge of managing extensive OCT input parameters and addressing unbalanced datasets. A review by Uddin et al. highlighted the prevalent use of the SVM algorithm in disease prediction studies, yet identified the RF classifier as achieving the highest accuracy across various applications, further supporting its effectiveness in predictive modelling [34]. 

A recent study by Lachance et al. explored the feasibility of using deep learning to improve predictions of VA improvement after MH surgery [12]. They developed a hybrid model, combining a deep learning convolutional neural network trained on preoperative high-definition OCT B-scans of the macula with a logistic regression model incorporating preoperative clinical features. The study found that while both clinical data and OCT-based models could independently predict postoperative VA improvements, integrating them into a hybrid model did not significantly enhance predictive accuracy. Consequently, future studies using AI for MH outcomes prioritized preoperative OCT parameters for predicting visual and anatomical results [4, 10, 29]. In the present study, the RF regressor identified MH closure status on OCT as the most critical predictor of postoperative VA outcomes. This finding underscores that eyes undergoing MH surgery are likely to show VA improvement if a normal foveal contour, intact outer retinal layers, and a healthy foveal RPE are achieved through successful MH closure.

In our study, the RF model demonstrated predictive performance for VA, accurately forecasting VA within 0.1 logMAR units in 61% of cases, within 0.2 logMAR units in 78%, and within 0.3 logMAR units in 87%. These results highlight the model’s potential for clinical application in prognosticating post-surgical outcomes. Enhancing the model’s accuracy could be achieved by incorporating a larger dataset with a greater number of preoperative OCT scans and MH cases during the training phase, thereby improving its generalizability and clinical utility.

This study provides valuable clinical insights into predicting visual outcomes in patients undergoing MH surgery. A dataset comprising 18,315 data points derived from 1,665 OCT images of 158 eyes treated surgically for MH was utilized to train, test, and compare the performance of six supervised ML algorithms. The ML model was developed using preoperative OCT data, which was meticulously graded by two independent graders, with inter-grader agreement validated to ensure the reliability of the annotations. To minimize variability in factors potentially influencing surgical outcomes, such as differences in surgeons, intraoperative dye usage, ILM peeling techniques, types of endotamponade, face-down positioning protocols, and surgery duration, the study focused on procedures performed by a single surgeon using a standardized surgical technique. This approach ensured a consistent evaluation of both anatomical and functional outcomes, enhancing the reliability of the findings. To our knowledge, this represents one of the first ML-based studies in MH with such a robust dataset. The findings underscore the potential of predictive models as adjunctive tools, aiding the clinician in postoperative prognosis and surgical decision-making. The study’s results are clear, clinically relevant, and readily applicable in practice.

Furthermore, the AI-based predictions for MH outcomes reported in this study have the potential to significantly influence clinical decision-making and enhance patient management strategies. By providing accurate prognostic information, these models can assist clinicians in setting realistic expectations for post-surgical visual outcomes, enabling more informed discussions with patients. Furthermore, AI-driven insights could aid in tailoring surgical approaches to individual patient profiles, optimizing outcomes by identifying cases that are more likely to benefit from specific techniques or interventions. Integrating these predictive tools into routine clinical workflows could also support early identification of cases requiring closer follow-up or adjunctive therapies, thereby improving overall patient care. Ultimately, the adoption of AI-based predictive models in the management of MH could lead to a more personalized, data-driven approach to ophthalmic care, improving both clinical efficiency and patient satisfaction. These advancements underscore the transformative role of AI in bridging the gap between complex retinal imaging data and actionable clinical decisions.

The retrospective design of the study inherently limited its scope by focusing solely on surgical cases, thereby excluding insights into the natural progression of the disease. A notable limitation of this study lies in the generalizability of its AI model, as its applicability to datasets from different patient populations, surgeons, or surgical settings remains uncertain. Future studies should aim to incorporate diverse datasets encompassing various patient populations with MH, operated on by different surgeons employing varied surgical techniques. This would enable the development of a more robust and universally applicable AI model for predicting post-surgical MH closure and VA outcomes. Despite these limitations, the findings underscore the potential of AI in predicting post-surgical MH outcomes, demonstrating its promising applicability even in routine clinical practice.

In conclusion, supervised ML algorithms, such as the RF regressor, can accurately predict visual outcomes following vitreoretinal surgery for MHs. These predictions can reassure patients with a low risk of failure and encourage consideration of advanced surgical techniques in more challenging cases. However, further validation studies are necessary before this model can be reliably integrated into clinical practice for forecasting MH surgical outcomes.

## Data Availability

No datasets were generated or analysed during the current study.

## Declarations

### Animal research

This article does not contain any studies with animals performed by any of the authors.

## Abbreviations

ML: Machine Learning
OCT: Optical coherence tomography
VA: Visual acuity
RF: Random Forest
SVM: Support vector machine
MH: Macular hole
ERM: Epiretinal membrane
ILM: Internal limiting membrane
RPE: Retinal pigment epithelium
ELM: External limiting membrane
AI: Artificial intelligence
LOGMAR: Logarithm of minimum angle of resolution
MSE: Mean square error
BBG: Brilliant Blue G
OOB: Out-of-bag
